# Effect of physical activity intervention on insulin resistance and appendicular body composition in gestational diabetes mellitus

**DOI:** 10.1038/s41598-026-35036-0

**Published:** 2026-01-22

**Authors:** Savni Apte, Preetha Ramachandra, Shyamala Guruvare, Shashikala K. Bhat, Saritha Kamath U, G. Arun Maiya

**Affiliations:** 1 Centre for Podiatry & Diabetic Foot Care and Research, Department of Physiotherapy, Manipal College of Health Professions, Manipal Academy of Higher Education, Manipal, 576104 Karnataka India; 2 Department of Physiotherapy, Manipal College of Health Professions, Manipal Academy of Higher Education, Manipal, 576104 Karnataka India; 3 Department of Obstetrics and Gynaecology, Kasturba Medical College, Manipal, Manipal Academy of Higher Education, Manipal, 576104 Karnataka India; 4 Department of Obstetrics and Gynaecology, Melaka Manipal Medical College (Manipal Campus), Dr. TMA Pai Hospital, Udupi, Manipal Academy of Higher Education, Manipal, 576104 Karnataka India; 5 Department of Medical Laboratory Technology, Manipal College of Health Professions, Manipal Academy of Higher Education, Manipal, 576104 Karnataka India

**Keywords:** Insulin resistance, Gestational diabetes mellitus, Physical activity intervention, Body composition, Endocrinology, Health care

## Abstract

The prevalence of gestational diabetes mellitus (GDM) is increasing worldwide, and a further increase is expected in the coming years. One of the common risk factors for GDM is reduced physical activity (PA), leading to impaired glycemic control and increased insulin resistance (IR). Hence, the objective of the study was to determine the effects of PA intervention on IR and appendicular body composition in individuals with GDM. After screening 135 participants, 52 were included based on the selection criteria. The eight-week PA intervention and an educational booklet with information about GDM and PA intervention were administered to the participants. A detailed analysis of the outcome measures was conducted at baseline and postintervention, including Homeostatic Model Assessment for Insulin Resistance (HOMA-IR) and appendicular body composition via body impedance analysis. There was a significant decrease in the HOMA-IR value (MD = 0.123, *p* < 0.05) and upper extremity (MD = 0.4, *p* < 0.05), and lower extremity (MD = 0.12, *p* < 0.05) fat mass‒muscle mass index. The eight-week PA intervention effectively improved insulin resistance and the appendicular body composition among pregnant women with GDM.

**Supplementary Information:**

The online version contains supplementary material available at 10.1038/s41598-026-35036-0.

## Introduction

“Gestational diabetes mellitus (GDM) is any degree of glucose intolerance with onset or first recognition during pregnancy.“^1^ The estimated global prevalence of GDM in 2019 was 10%, which increased to 14% in 2022 and is expected to increase in the coming years^2^. Increased insulin resistance (IR) is a normal phenomenon during pregnancy in which insulin-mediated glucose transport is reduced by 50% in normal pregnancy and is compensated by a 200–250% increase in insulin secretion to maintain euglycemia^3^. The role of placental hormones as insulin antagonists causes increased IR during pregnancy^4^. On the other hand, non-modifiable risk factors, such as a positive family history of diabetes mellitus (DM) or a history of polycystic ovarian syndrome (PCOS), and modifiable factors, such as physical inactivity, pose additional risk for the development of GDM^5^.

The worldwide prevalence of GDM has increased from 8.9% in 2011 to 23.7% in 2020^6^. The rise in obesity rates leads to an increased prevalence of GDM^7^. High pre-pregnancy body mass index (BMI) and waist circumference are also associated with the development of GDM^8^. Women with GDM were found to have a significantly high fat mass-to-muscle mass index^9^. Higher muscle mass improves insulin sensitivity, as muscles play a significant role in glucose uptake^10^. Increased body fat was found to be positively associated with increased chances of cesarean section and adverse fetal outcomes^11^. Increased insulin resistance was also found to affect anthropometric fetal indices, such as fetal macrosomia, as well as increased subcutaneous fat thickness in newborns of individuals with gestational diabetes mellitus^12,13^.

The American College of Obstetrics and Gynecology recommends engaging in at least 150 min of moderate-intensity PA weekly during pregnancy^14^. Physical inactivity poses an additional risk for facilitating IR by increasing β-cell insufficiency, oxidative damage, mitochondrial dysfunction, and inflammation^15^. In a study by Sun et al. (2021), it was stated that PA levels were lower in 60% of pregnant women during the preconception period, resulting in increased GWG^16^. The feelings of tiredness, low energy, lack of support, and family members discouraging participation in moderate intensity PA are a few of the commonly stated barriers to performing regular PA during pregnancy^17^.

Previous studies have specified a few common barriers to incorporating regular PA, such as household work commitments and a lack of time^18^. The difficulty in maintaining the continuity of an exercise program at home due to a lack of equipment was emphasized in a recent study by Zhao et al. (2022)^19^. Yaping et al. (2022) also stated the lack of potential outcome measures, such as the evaluation of insulin resistance, as one of the limitations of the study^20^.

Despite the increasing prevalence of GDM in India, there is a dearth of literature determining the effects of PA intervention on IR and body composition. Hence, the objective of the present study was to determine the effects of physical activity intervention on IR and appendicular body composition among individuals with GDM.

## Methods

### Sample size calculation

The sample size was calculated to obtain the mean difference of 6.9 mg/dL in fasting blood glucose^21^and a standard deviation of 11.8; the required sample size was 46, and with a 10% attrition rate, the final sample size was 52.

#### Study design

The present study was a feasibility trial, a pre-post interventional study. The study commenced after receiving clearance from the Institutional Ethics Committee (IEC1: 86/2022). The study was registered in the Clinical Trial Registry-India on 07/07/2022 (CTRI/2022/07/043832). The present study was conducted in accordance with the Declaration of Helsinki. The manuscript aligns with the recommendations for the conduct, reporting, editing, and publication of scholarly work in medical journals. This was a feasibility trial involving a single-arm interventional design with a convenience sampling method conducted at the obstetric outpatient departments of a hospital in the southern coastal region of India. The participant information sheets were given to all the participants, and a detailed explanation of the study was provided. The participants were enrolled in the study after obtaining signed informed consent. The duration of the study was from July 2022 to July 2023.

### Procedure

Participants visiting the obstetric department of the hospital were screened based on specific inclusion and exclusion criteria. Their demographic information was recorded, including age, gestational age at the time of enrollment, family history of diabetes mellitus, and self-reported pre-pregnancy weight. Additionally, details regarding medications, such as the use of oral hypoglycemic agents (OHA) and their dosages, were documented. Information on whether a diet was prescribed by a dietitian or if participants followed a self-modified diet was also noted.

Anthropometric characteristics, such as height and weight, were assessed to calculate the BMI at the time of enrollment in the study. The height was assessed using a wall-mounted stadiometer, and weight was assessed using an Omron Karada scan machine, which was also used to assess body composition. The information regarding gestational age at delivery, the term of pregnancy, BMI assessed before delivery, and birth weight was retrieved from the hospital database.

The physical activity intervention was administered to the participants for eight weeks along with a validated booklet containing details of GDM and PA intervention. The procedure of development, validation, and pilot testing of the booklet is published elsewhere^22^. The PA intervention included in-person sessions conducted once in two weeks, education and counselling, progression of the PA intervention during in-person sessions, once-a-week motivational phone calls, and adherence checks with an exercise diary. All the outcomes, including IR and body composition, were assessed at baseline and after 8 weeks.

### Participants

The study included pregnant women who were diagnosed with GDM according to the International Association of Diabetes and Pregnancy Study Groups (IADPSG)^23^ diagnostic criteria. Participants aged between 18 and 45 years, with a gestational age (GA) of less than 28 weeks, able to speak and understand English or Kannada, with a PA level of less than 600 MET min per week, assessed using the Global Physical Activity Questionnaire (GPAQ)^24^ and planning to continue receiving consultation from the same hospital until delivery was included in the study. Participants with GDM with any complications observed during previous scans, systemic issues such as obstructive or restrictive respiratory diseases, cardiovascular diseases, cognitive and mental health issues, or any condition that would impede their participation in the PA program, and those who declined to participate in the study were excluded.

### Physical activity intervention

The PA intervention was conducted and reported as per the Template for Intervention Description and Replication (TIDieR) checklist^25^. The primary investigator (PI) administered and provided progression in the PA intervention. The PI has a master’s degree in physiotherapy and is certified in prenatal yoga. In-person sessions were held with participants every other week, coinciding with their routine visits to the obstetrics department. During these sessions, participants were encouraged to practice the recommended physical activities (PA) at home by using a provided booklet until their next appointment with the PI. Each in-person session included a progression of activities. A total of four in-person sessions were conducted over the duration of 8 weeks of the study.

#### Education and counseling

All the participants were educated regarding the concept of GDM, its causes, risk factors, guidelines to follow the PA intervention, and precautions to be taken while following the PA intervention. Additionally, various methods were taught for the self-assessment of PA intensity at home, such as the heart rate evaluation and using the heart rate maximum formula (HR_max_ = 220-age), and the Rating of Perceived Exertion (RPE) scale. They were also informed regarding all the contraindications to follow up with PA intervention and the indications for visiting the obstetrician.

#### Posture corrections and other ergonomics

The importance of correct posture was explained to the participants. They were taught how to correct and maintain proper posture while sitting and standing. Additionally, instructions were provided on pillow arrangements and sleeping positions.

#### Breathing exercise/pranayama and yoga Nidra

Breathing techniques like nadi-shodhana & bhramari pranayama, deep breathing exercises, thoracic expansion exercises, meditation, and yoga nidra (a relaxation technique given in yoga) were administered and taught to all the participants. The participants were instructed to practice the activities daily for at least 15 min.

#### Strength training

The strength training involved strengthening the major muscle groups of the upper and lower extremities. The 10 Repetition Maximum (10 RM) is the maximum weight a person could lift only 10 times, which was assessed during the in-person session with the participants via the direct method. The weights were given progressively until the participants reached the maximum weight with which they could perform for only 10 repetitions. Exercises were given and progressed by increasing the number of sets of 100% of the 10 RM, followed by 75% of the 10 RM, and then 50% of the 10 RM.

#### Aerobic training

Aerobic training included brisk walking, which can be easily adapted to home settings. During the first four weeks, the intensity of the aerobic activities was maintained at 55% to 65% of the HRmax or a RPE of 9 to 11. This intensity was increased from the 5th week to the 8th week to an RPE between 12 and 14 (somewhat hard), corresponding to 64% to 76% of their HR_max_. The total duration of the activity ranged from 30 to 35 min.

#### Pregnancy-specific activities

*Pelvic floor exercises/Kegel exercises:* Pelvic floor exercises, i.e., Kegel’s exercises with contract-hold-relax technique and rapid contract-relax technique, were administered in the side-lying position. Participants were instructed to perform a minimum of 25 contractions per set and 4–6 sets throughout the day to complete at least 100–150 contractions of the pelvic floor muscles per day.

*Prenatal yoga:* Warm-up includes all joints active movements, gentle spinal side bends and sideways twists, chair and mat yoga-asanas, including virabhadrasana-1, and 2, titli asana, chakki chalasana, and malasana.

The intensity of pregnancy-specific activities was maintained between 8 and 9 on the RPE scale. Participants were instructed to perform pregnancy-specific activities at least five days a week since they were mild-intensity activities.

*Labor positions for pain relief:* Participants were taught pain relief and birthing positions, such as the on-all-four position and forward-leaning, during the last week of the intervention program.

#### Active sitting time

Participants were encouraged to use 0.5 to 1 L water bottles as weights for upper extremity strength training. Additionally, performing dynamic quadriceps without weights, ankle toe pumps, intrinsic foot muscle exercises, core muscle exercises, and Kegel’s exercises were encouraged during leisure sitting time.

#### Educational material and diary

A 20-page information booklet, containing all the necessary information, pictorial presentations of the activities, and gestational weekwise PA interventions, was handed over to all participants as a reference copy and a diary to record their daily physical activities^26^.

#### Exercises to alleviate pain

Specific exercises, including strengthening and mild massage techniques, were taught to alleviate musculoskeletal pain and discomfort.

#### Involvement of the caregiver

A close relative was called for at least one of the early sessions, and all the activities were demonstrated. They were encouraged to help the participants perform activities at home.

#### Regular hospital care

Regular consultations with the doctor, regular nutritional supplements, and medications were continued for all the participants.

The detailed procedure of all the activities was provided in **Supplementary file 1**.

The PA intervention was customized for each participant, and progression was performed every 2 weeks. The progression was given in terms of the total duration of the intervention, repetitions, or holding time of each yoga pose. The therapist ensured that the PA level was maintained above 600 MET min per week throughout the study duration.

### Assessment of participant preferences

Participants were asked about their preference for PA during the first in-person session. They were asked to choose their preferred PA between strength training and aerobic training. Participants could also choose a combination of strength and aerobic training, along with pregnancy-specific activities.

### Outcome measures

The outcome measures included Homeostatic Model Assessment for Insulin Resistance (HOMA-IR), fasting insulin, fasting blood glucose (FBG) values, and body composition, including skeletal muscle and fat mass of the upper extremities (UE) and lower extremities (LE).

#### HOMA-IR

HOMA-IR evaluation was conducted with serum samples obtained after 8 to 10 h of fasting. The test was conducted in a biochemistry laboratory by professionals. The HOMA-IR value was calculated with the FBG and fasting insulin (FI) values.

#### Body composition

Body composition was measured via an Omron Karada body composition analysis machine. The whole-body composition included UE and LE skeletal and fat mass percentages. All the instructions for body composition analysis were followed as given in the user manual. The fat mass to muscle mass index was directly calculated by dividing fat mass by muscle mass in percentages to find the relative proportion between fat mass percentage and muscle mass percentage.

### Statistical analysis

The data were entered into an Excel sheet and imported into Jamovi version 2.6.44. The normality of the outcomes was assessed using the Shapiro-Wilk test. The normally distributed quantitative outcomes were reported in mean and standard deviation, and non-normally distributed data were reported in median and interquartile range. The categorical data were assessed using a binomial test and reported in percentages. The correlation matrix was used to assess the correlation between birth weight and insulin resistance. Preinterventional and postinterventional outcomes were compared via the Wilcoxon signed-rank test. The level of significance was set at *p* < 0.05.

## Results

**Fig. 1:**
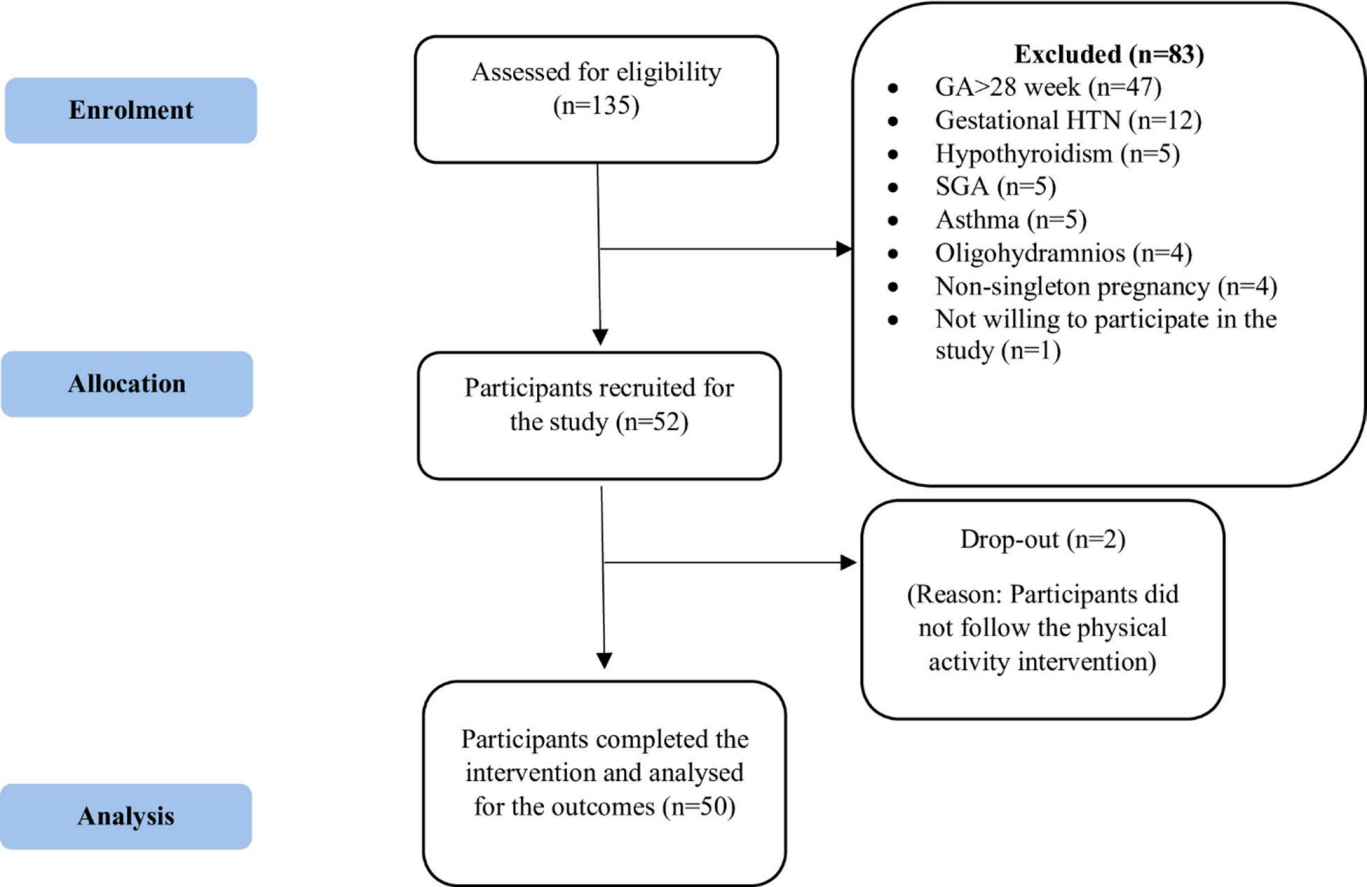
CONSORT participant flow diagram. Abbreviations: GA, gestational age; SGA, short for gestational age; HTN, Hypertension.

A total of 135 participants were screened via the Global Physical Activity Questionnaire (GPAQ); 52 were included in the study based on the inclusion criteria, and 83 were excluded for various reasons. A total of 50 participants completed the study intervention, and two participants were considered to have dropped out due to noncompliance with the PA intervention **(**Fig. 1**).**

### Participant characteristics

The participants’ ages ranged from 21 years to 40 years, and the GA ranged from 23.3 weeks to 28 weeks. Only 6 of the 50 participants followed a structured diet prescribed by a dietician; the others consumed a self-modified, low-sugar diet. A total of nine participants had a BMI above 30 kg/m², 16 participants had a BMI between 25 and 30 kg/m², 25 participants were within the normal range of BMI, and none were underweight^27^ **(**Table 1**).** Out of 50 participants, 13 were prescribed OHA. Among them, nine participants were prescribed a dosage of 500 mg to be taken once a day, and 500 mg twice a day for 4 participants. The gestational age (GA) at delivery ranged from 36 weeks to 40.1 weeks. The term of pregnancy was defined and classified according to the guidelines by the World Health Organization (WHO) and the American College of Obstetrics and Gynecology^28,29^. Birth weight was classified according to WHO guidelines^30^. The two newborns with low birth weights were born in late preterm, i.e., between 36 and 37 weeks of gestation.

**Table 1:** Demographic and baseline clinical characteristics of the participants.

Sr no	Outcomes	Values
1	Age (years)	27 (5.75)
2	Gestational age (weeks)	27.1 (1.17)
3	Gestational age at delivery (weeks)	38 (1.8)
4	Term of pregnancy (%)	Later preterm (36–37 weeks): 10 Early term (37–38.6 weeks): 60 Full term (39–40.6 weeks): 28 Late term (41-41.6 weeks): 2
5	Family history of diabetes mellitus (%)	Yes: 32 No: 68
6	Pre-pregnancy weight	60 (8.27)
7	Diet prescription (%)	Prescribed by a dietitian: 12 Self-modified diet: 88
8	Pre-pregnancy BMI (Kg/m^2^)	23.8 ± 4.29
9	BMI at the time of enrolment (Kg/m^2^)	26.6 ± 4.6
10	BMI before delivery (Kg/m^2^)	27.5 ± 3.8
11	Gestational weight gain (Kg)	9.28 ± 2.99
12	Participants on OHA (%)	Yes: 26 No: 74
13	HOMA-IR	2.26 ± 0.353
14	FBG (mg/ dL)	93 (4.75)
15	FI (µu/mL)	9.39 (1.78)
16	Fat mass percentage of UE (%)	41.5 ± 8.7
17	Fat mass percentage of LE (%)	43.4 ± 5.8
18	Muscle mass of UE (%)	32.8 (9.05)
19	Muscle mass of LE (%)	36.6 ± 3.6
20	UE fat mass/ muscle mass index	1.31 (0.491)
21	Lower extremity fat mass/ muscle mass index	1.19 ± 0.17
22	Birth weight (grams)	3039 ± 346
23	Birth weight classification (%)	Low birth weight (< 2500 g): 4 Normal birth weight (2500–4000 g): 96
24	Level of physical activity (MET min per week)	480 (100)

Abbreviations: Sr no, serial number; BMI, body mass index; OHA, oral hypoglycaemic agents; HOMA−IR, homeostatic model assessment for insulin resistance; FBG, fasting blood glucose; FI, fasting insulin; UE, upper extremity; LE, lower extremity; MET, metabolic equivalent of task.

The values are provided in mean and SD for normally distributed data, in median and IQR for skewed data, and categorical variables are stated in percentages.

### Effect of PA intervention on HOMA-IR, FI, and FBG

**Fig. 2:**
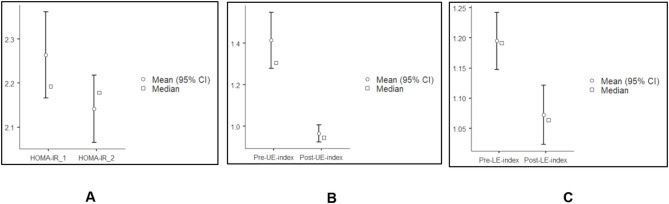
Effect of physical activity intervention on. (**A**) Pre-post change in HOMA-IR***, (**B**) Pre-post changes in upper extremity fat mass- muscle mass index***, (**C**) Pre-post changes in lower extremity fat mass- muscle mass index***. Abbreviations: CI, confidence interval; Pre-UE- index, pre-intervention upper extremity fat mass-muscle mass index; post-UE-index, post-intervention upper extremity fat mass-muscle mass index; Pre-LE- index, pre-intervention lower extremity fat mass-muscle mass index; post-LE-index, post-intervention lower extremity fat mass-muscle mass index. (Source: Jamovi version 2.4.8).

HOMA-IR (MD = 0.12, *p* = 0.006) and FBG (MD = 6.5, *p* < 0.001) were reduced significantly post-intervention **(**Fig. 2**)**. The FI levels were slightly increased post-intervention (MD = 0.2, *p* = 0.26) **(**Table 2**)**.

### Effect of PA intervention on body composition

There was a significant reduction in UE (MD = 3.05, *p* < 0.001) and LE (MD = 3.05, *p* < 0.001) fat mass percentage and an increase in muscle mass percentage in UE (MD = 2.15, *p* < 0.001) and LE (MD = 1.45, *p* < 0.002) postintervention **(**Table 2**)**. The UE (MD = 0.39, *p* < 0.001) and LE (MD = 0.11, *p* < 0.001) fat mass‒muscle mass indices decreased significantly after eight weeks of PA intervention **(**Fig. 2**)**.

**Table 2:** Effect of physical activity Intervention.

Sr no	Outcome	Pre-intervention (Median and IQR)	Post-intervention (Median and IQR)	Mean difference	95% confidence interval	Effect size	Timepoints Comparison (*p*-value)
1	HOMA-IR	2.19 (0.43)	2.18 (0.37)	0.12	(0.04, 0.2)	0.4	0.006**
2	FBG (mg/ dL)	93 (4.75)	86.5 (5)	6.5	(5, 8.5)	0.8	< 0.001***
3	FI (µu/mL)	9.39 (1.78)	10.3 (1.84)	0.2	(0.5, 0.1)	0.18	0.26
4	Fat mass of upper extremity (%)	40.3 (12.3)	38.2 (10.5)	3.05	(2.5, 3.6)	1	< 0.001***
5	Fat mass of lower extremity (%)	42.3 (8.45)	39.4 (8.28)	3.05	(2.5, 3.65)	1	< 0.001***
6	Muscle mass of upper extremity (%)	31.1 (10.6)	34.4 (10.7)	2.15	(2.8, 1.2)	0.6	< 0.001***
7	Muscle mass of lower extremity (%)	36.4 (4.52)	37.8 (5.35)	1.45	(2.2, 0.7)	0.5	0.002**
8	Upper extremity fat mass/ muscle mass index	1.31 (0.4)	0.9 (0.1)	0.39	(0.2, 0.5)	0.8	< 0.001***
9	Lower extremity fat mass/ muscle mass index	1.19 (0.17)	1.06 (0.2)	0.11	(0.09, 0.1)	0.9	< 0.001***
10	Physical activity level (MET min per week)	480 (100)	880 (190)	420	460, 370	1	< 0.001***

Abbreviations: Sr no, serial number; IQR, inter−quartile range; HOMA−IR, Homeostatic Model Assessment for Insulin Resistance; FBG, Fasting Blood Glucose; FI, Fasting Insulin; MET, metabolic equivalent of task.

* *p*< 0.05,

**p<0.01.

***p<0.001.

### Correlation between IR and birth weight

**Fig. 3:**
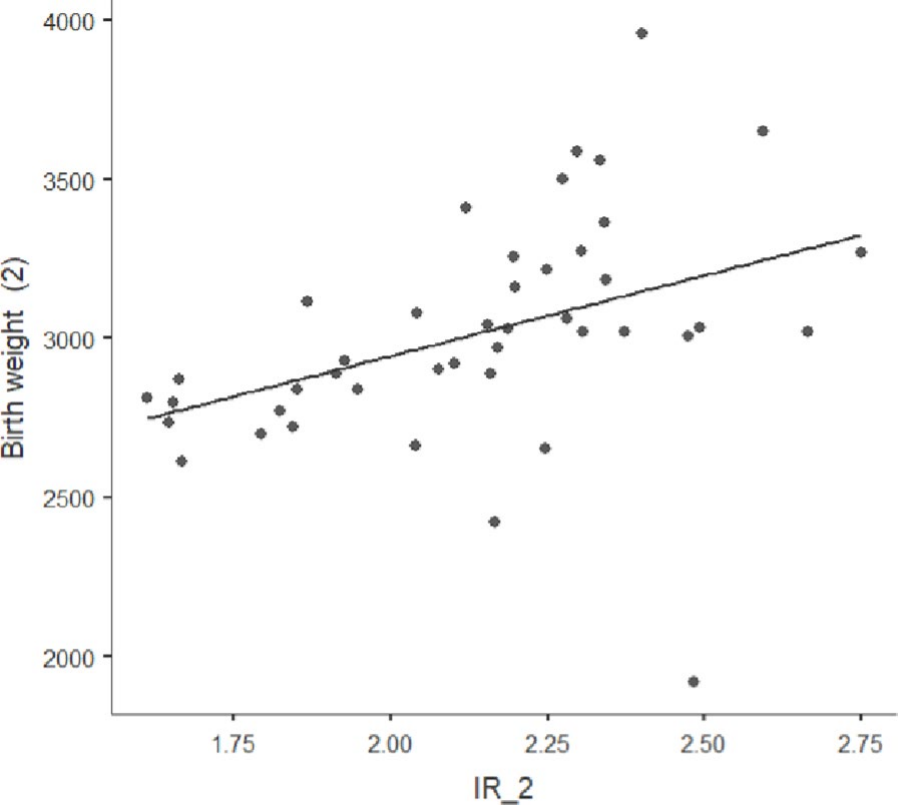
Correlation of birth weight to post-interventional insulin resistance. Abbreviations: IR_2, Post−interventional Insulin resistance; Birth weight, birth weight of the newborn. (Source: Jamovi version 2.4.8).

The birth weights of the newborns ranged from 1920 to 3960 g. Two newborns had low birth weights, i.e., ranging between 1500 and 2500 g. None had very low birth weights (< 1500 g) or high birth weights (> 4000 g). A moderate positive correlation was obtained between IR and the birth weight of newborns (*r* = 0.42, *p* = 0.002) **(**Fig. 3**)**.

### Correlation between change in insulin resistance with weight change and fat mass changes

A partial correlation model was used in regression analysis to examine the relationship between changes in IR and GWG, as well as fat mass changes, controlling for covariates such as age and pre-pregnancy weight. The Spearman correlation coefficient was used as the data was not normally distributed. A weak negative correlation was obtained between IR changes and GWG (ρ = 0.243, *p* = 0.09). A weak positive correlation was obtained between IR changes and fat mass changes (ρ = 0.257, *p* = 0.07). No correlation was observed between GWG and fat mass changes (ρ = 0.038, *p* = 0.8).

### Adherence and adverse events

Adherence was checked based on the participants’ self-reported diaries. Out of 52 participants, 50 were compliant with the PA intervention. The criterion for adherence was to complete at least 150 min of moderate-intensity PA in a week, which is approximately 600 MET per week. Thus, the obtained compliance with the study was very high (96.15%). No adverse events were observed throughout the study.

## Discussion

The present study aimed to determine the effects of physical activity intervention on the IR and appendicular body composition among pregnant women with GDM. An increased level of PA significantly improved IR and appendicular body composition **(**Table 2**)**.

There was a slight increase in FI but a significant decrease in FBG, which reduced HOMA-IR levels **(**Table 2**)**. PA has an insulin-sensitizing effect by increasing the amount of GLUT4 and its translocation, increasing the influx of glucose molecules into cells^31^. These results are in line with those of a previous study in which exercise was implemented on pregnant women at risk of GDM, which revealed significant decreases in FI, FBG, and HOMA-IR levels^32^. Conversely, in a study by Garnæs et al. (2016), the researchers administered an exercise program to pregnant women with obesity and found no effect of exercise on HOMA-IR values, which could be due to obesity^33^. In the present study, the influence of non-modifiable risk factors, such as positive family history of diabetes mellitus DM and genetic predisposition, could be the reason for increased fasting insulin levels post-intervention.

The therapist prescribed the preferred physical activity to maintain a minimum of 600 MET minutes per week, equivalent to 150 min of moderate-intensity activity weekly. The PA intervention involved strength training, aerobic activities, and pregnancy-specific activities, which may have improved the appendicular body composition. The significant decrease in the index indicates a significant decrease in postinterventional fat mass and an increase in muscle mass. The results obtained in the present study are in line with the previous study, which reported that regular exercise prevents excessive weight gain during pregnancy^34^. A survey by Andersson-Hall et al..(2021) also revealed that pregnant women involved in strength training experienced less fat mass gain than those not following exercise^35^.

The obtained compliance with the study was 96.16%, which is considered high compliance. The possible reasons for the obtained compliance in the present study could be because of once-a-week in-person sessions with the therapists, education, and counselling of the participants, which might have improved their understanding of the need for exercise during pregnancy. Additionally, motivational calls and provision of the booklet as a reference material could have improved the confidence in participants to practice recommended activities at home.

Among the 50 participants, 38 expressed a preference for a combination of strength training, brisk walking, and pregnancy-specific activities, while 12 opted to focus solely on strength training and pregnancy-specific exercises. Although 38 participants initially chose the combination of activities, 35 ultimately engaged only in strength training and pregnancy-specific exercises, supplemented by slow-paced walking. Participants reported a heightened fear of falling when attempting to walk faster than their usual pace. This increased fear of falling specifically during late pregnancy was also reported in a recent study by Wang et al. (2025)^36^.

Although determining a correlation was not the objective of our study, we found a moderate correlation between IR and the birth weight of newborns **(**Fig. 3**)**. The underlying pathophysiology may be that maternal diabetes, which facilitates fetal fat deposition, increases the incidence of large for gestational-age newborns^37^. Non-significant correlations were obtained between IR changes, GWG, and fat mass changes, as numerous factors contribute to the GWG besides fat mass, such as the weight of the fetus and fluid accumulation in the body. The intervention was initiated in the third trimester; thus, the GWG could be higher in the first two trimesters compared to the third trimester.

The weak correlation between IR changes and fat mass changes could be because IR can be influenced by other factors, such as dietary habits and psychosocial components, which were not controlled in this study. Similar results were obtained in a study by Nasis et al. (2005) in which there was a change in insulin sensitivity without changes in fat mass and body weight, signifying that changes in body composition may not directly impact IR^38^.

The strength of the present study was that specific and sensitive outcomes, including FI, IR, FBG, and body composition, were evaluated. A validated physical activity promotion program booklet and a diary were given to all the participants to note the daily physical activities performed, improving adherence to the given PA intervention. We also emphasized PA, which can be performed while sitting, to convert sitting time to an active period.

In the present study, we did not note the participants’ diet, stress levels, or psychosocial aspects contributing to hyperglycemia during pregnancy. Future studies can include randomized controlled trials that consider diet and other psychosocial aspects and provide comprehensive rehabilitation to participants. Due to increased fluid accumulation in the trunk area during the third trimester of pregnancy, assessment of segmental fat and muscle mass analysis was recommended in the pregnant population^39^. Thus, in the present study, we assessed and reported only segmental body composition and did not report fat mass and muscle mass values in the trunk areas due to the possibility of inaccuracies. However, the assessment of body composition with advanced technology such as DEXA scan could be done in future studies to avoid possible inaccuracies.

### Clinical implications

PA offers many options according to the patient’s choice, including strength training, aerobic training, yoga, and pelvic floor exercises, which may break the monotony of performing the same exercises every day. An educational booklet for promoting PA among pregnant women can be used as reference material as a part of the intervention, which increases confidence among participants to perform activities at home and may improve adherence. We used phone calls and messages to motivate participants and a diary to track the PA given to all the participants, which can also be used in clinical and hospital settings.

## Conclusion

The PA intervention administered for eight weeks effectively improved the IR and appendicular body composition in pregnant women with GDM. Our study reinforces the potential of PA intervention in managing GDM.

## Supplementary Information

Below is the link to the electronic supplementary material.

Supplementary Material 1

## Data Availability

The authors confirm that the data supporting the findings of this study will be available on request by contacting the corresponding author (Dr. G Arun Maiya, [arun.maiya@manipal.edu](mailto: arun.maiya@manipal.edu) ).
