# Supplementary material for: Effect of physical activity intervention on insulin resistance and appendicular body composition in gestational diabetes mellitus
Source: Sci Rep. 2026 Jan 22;16:5469. doi: 10.1038/s41598-026-35036-0 (PMC12886755; doi:10.1038/s41598-026-35036-0)
Supplement: Supplementary file 1 — Supplementary Material 1 [file 41598_2026_35036_MOESM1_ESM.docx]

**Title: Effect of physical activity intervention on insulin resistance and appendicular body composition in gestational diabetes mellitus**

**Supplementary file 1: Physical activity intervention**

***Posture corrections and other ergonomics:*** The importance of correct posture was explained to the participants. They were taught how to correct and maintain proper posture while sitting and standing. Additionally, instructions were provided on pillow arrangements and sleeping positions. Participants were encouraged to consciously maintain a neutral pelvic tilt and to avoid bending from their backs; instead, they were told to use squats to pick up lightweight objects from the floor. Other helpful tips included using a footstool for support while sitting in a chair and placing a small pillow for lower back support.

***Breathing exercise/pranayama and yoga nidra:***

Breathing techniques like nadi-shodhana & bhramari pranayama, deep breathing exercises, thoracic expansion exercises, meditation, and yoga nidra (a relaxation technique given in yoga) were administered and taught to all the participants. In Nadi-shodhana pranayama, the participants were instructed to sit in a comfortable position (with or without back support) and close the right nostril and inhale through the left nostril, then close the left nostril and exhale through the right nostril, and then repeat vice versa. Participants were instructed not to hold their breath throughout the procedure. In the Bhramari pranayama technique, participants were instructed to sit in a comfortable position and were told to close their ears with their thumbs and inhale through the nose and make a humming sound while exhaling through the nose.

The deep breathing exercise included inhaling through the nose and exhaling through the mouth by pursing the lips. The thoracic expansion exercises included inhaling while flexing the shoulders and exhaling while bringing the arms back to the starting position. In the yoga nidra technique, the participants were instructed to be in the side-lying position and shift their focus from each segment and joint of the body, and relax each segment simultaneously. The participants were instructed to practice the activities daily for at least 15 minutes.

***Strength training:***

The strength training involved strengthening the major muscle groups of the upper and lower extremities. The 10 Repetition Maximum (10 RM) is the maximum weight a person could lift only 10 times, which was assessed during the in-person session with the participants via the direct method. The weights were given progressively until the participants reached the maximum weight with which they could perform for only 10 repetitions. Exercises were given and progressed by increasing the number of sets of 100% of the 10 RM, followed by 75% of the 10 RM, and then 50% of the 10 RM.

***Aerobic training:***

Aerobic training included brisk walking, which can be easily adapted to home settings. During the first four weeks, the intensity of the aerobic activities was maintained at 55% to 65% of the HRmax or a RPE of 9 to 11. This intensity was increased from the 5^th^ week to the 8^th^ week to an RPE between 12 and 14 (somewhat hard), corresponding to 64% to 76% of their HR_max_. The total duration of the activity ranged from 30 to 35 minutes.

***Pregnancy-specific activities:***

*Pelvic floor exercises/Kegel exercises:* Pelvic floor exercises, i.e., Kegel’s exercises with contract-hold-relax technique and rapid contract-relax technique, were administered in the side-lying position. Participants were instructed to perform a minimum of 25 contractions per set and 4-6 sets throughout the day to complete at least 100-150 contractions of the pelvic floor muscles per day.

*Prenatal yoga:* Warm-up includes all joints active movements, gentle spinal side bends and sideways twists, chair and mat yoga-asanas, including virabhadrasana-1, and 2, titli asana, chakki chalasana, and malasana.

Virabhadrasana-1 and 2: For virabhadrasana-1, participants were instructed to stand upright and step the right foot forward into a lunge, positioning the knee directly above the ankle, with the left foot angled outward approximately 45 degrees. They were then guided to rotate their hips forward, aligning shoulders and hips to face the front leg, and raise both arms overhead with palms facing inward, extending upward through the fingertips. Participants were told to maintain this posture for 10-15 seconds and repeat it 5 to 7 times, then repeat the procedure on the opposite side. In Virabhadrasana-2, along with the same placement of feet and trunk, they were directed to extend their arms horizontally at shoulder height, parallel to the ground, with palms facing downward.

Titli asana: Participants were instructed to sit on the floor with their legs extended forward. They were then guided to bend both knees and bring the soles of the feet together, drawing the heels as close to the pelvis as comfortably possible. While keeping the spine erect, participants were asked to hold their feet with both hands. They were then instructed to gently move the knees up and down in a flapping motion, resembling the wings of a butterfly. This movement was performed rhythmically for the prescribed duration, maintaining a relaxed and steady breathing pattern throughout.

Chakki chalasana: Participants were instructed to sit on the floor with their legs extended straight and spread wide apart. They were guided to interlock the fingers of both hands and extend the arms forward at shoulder height. Keeping the spine erect, participants were instructed to begin rotating the upper body in a circular motion, as if churning a mill, by moving the interlocked arms in a large circular path over the legs. The movement was performed in a clockwise direction for 8 to 10 rounds, followed by the same number of rotations in the counterclockwise direction. Throughout the practice, participants were asked to maintain steady breathing and avoid bending their knees.

Malasana: Participants were instructed to stand with their feet slightly wider than hip-width apart. They were then guided to slowly bend the knees and lower the hips into a deep squat, ensuring that the feet remained flat on the floor and the spine was kept upright. Participants were asked to bring their palms together in a prayer position (Namaste) at the chest and to press the elbows gently against the inner knees to help open the hips. They were instructed to maintain a steady and relaxed breathing pattern while holding the position for 10 seconds and repeat it 5 to 7 times.

The intensity of pregnancy-specific activities was maintained between 8 and 9 on the RPE scale. Participants were instructed to perform pregnancy-specific activities at least five days a week since they were mild-intensity activities.

*Labor positions for pain relief:* Participants were taught pain relief and birthing positions, such as the on-all-four position and forward-leaning, during the last week of the intervention program.

***Active sitting time:*** Participants were encouraged to use 0.5 to 1L water bottles as weights for upper extremity strength training, including biceps curls, triceps strengthening, shoulder flexion and abduction with weight, dynamic quadriceps, ankle toe pumps, intrinsic foot muscle exercises including picking the tissue with curled toes, core muscle exercises including abdominal tuck-in, gluteal activation and Kegel’s exercises while sitting.

***Educational material and diary:*** A 20-page information booklet, containing all the necessary information, pictorial presentations of the activities, and gestational weekwise PA interventions, was handed over to all participants as a reference copy and a diary to record their daily physical activities.

***Exercises to alleviate pain:*** Specific exercises, including static strengthening exercises, stretching, and mild massage techniques, were taught to alleviate musculoskeletal pain and discomfort.
